# Automatic Frequency Tuning Technology for Dual-Mass MEMS Gyroscope Based on a Quadrature Modulation Signal

**DOI:** 10.3390/mi9100511

**Published:** 2018-10-10

**Authors:** Jia Jia, Xukai Ding, Yang Gao, Hongsheng Li

**Affiliations:** 1 School of Instrument Science and Engineering, Southeast University, Nanjing 210096, China; 230169207@seu.edu.cn (J.J.); dingxukai@126.com (X.D.); gao-yang@seu.edu.cn (Y.G.); 2 Key Laboratory of Micro-Inertial Instruments and Advanced Navigation Technology, Ministry of Education, Nanjing 210096, China

**Keywords:** dual-mass MEMS gyroscope, frequency tuning, frequency split, quadrature modulation signal, frequency mismatch

## Abstract

In order to eliminate the frequency mismatch of MEMS (Microelectromechanical Systems) gyroscopes, this paper proposes a frequency tuning technology based on a quadrature modulation signal. A sinusoidal signal having a frequency greater the gyroscope operating bandwidth is applied to the quadrature stiffness correction combs, and the modulation signal containing the frequency split information is then excited at the gyroscope output. The effects of quadrature correction combs and frequency tuning combs on the resonant frequency of gyroscope are analyzed. The tuning principle based on low frequency input excitation is analyzed, and the tuning system adopting this principle is designed and simulated. The experiments are arranged to verify the theoretical analysis. The wide temperature range test (-20${}^{\circ }C$–60${}^{\circ }C$) demonstrates the reliability of the tuning system with a maximum mismatch frequency of less than 0.3 Hz. The scale factor test and static test were carried out at three temperature conditions (−20 ${}^{\circ }$C, room temperature, 60 ${}^{\circ }$C), and the scale factor, zero-bias instability, and angle random walk are improved. Moreover, the closed-loop detection method is adopted, which improves the scale factor nonlinearity and bandwidth under the premise of maintaining the same static performances compared with the open-loop detection by tuning.

## 1. Introduction

With the rapid development of MEMS (Microelectromechanical Systems) technology, silicon micromachined gyroscopes have attracted more attention. As a miniature sensor for measuring angular velocity, MEMS gyroscopes have been widely used in military and civilian fields [1,2,3,4]. Therefore, the performance requirements of the MEMS gyroscope are also increasing [2,5,6]. When the drive mode and sense mode of the gyroscope have the same resonant frequency (mode-matching), the gyroscope can have a higher signal-to-noise ratio of the output signal without deteriorating the circuit noise. The principle of operation of dual-mass MEMS gyroscopes is based on the Coriolis coupling between the two operating modes (drive and sense modes) when a rotation is applied about the sensitive axis of the device. Efficient energy transfer from the drive mode to the sense mode, which is largely determined by the frequency matching condition, is a principal factor in performance. In practice, however, fabrication imperfections and environmental variations are always present, resulting in a frequency mismatch between the two modes [3,5,7,8]. This frequency mismatch lead to degraded sensitivity, resolution, signal-to-noise ratio, and poor zero bias stability. Therefore, it is necessary to study the method of eliminating frequency split (Δf) so that the gyroscope is in the mode-matching condition.

There are several post-processing frequency tuning technologies to eliminate frequency split, such as local thermal stress technology [9,10], micromachining correction technology [11,12], and electrostatic adjustment technology [1,2,3,4,5,6,7,8,13,14,15,16,17,18,19]. In [9,10], the structural stress and material parameters of the gyroscope are changed by the heat generated by loading voltage, and the resonant frequency of the gyroscope is altered to realize the mode-matching. While frequency tuning by micromachining correction technology is achieved by changing the structural parameters of the gyroscope through polysilicon precipitation [11] or laser trimming [12]. The above two techniques share the same drawback of requiring, to some extent, a manual intervention, so that they are not desirable for mass production. Moreover, these two technologies can cause unstable output due to temperature changes and are not suitable for real-time adjustment of the resonant frequency.

A more effective method at present is the electrostatic adjustment technology, which utilizes a structure-specific electrostatic negative stiffness effect to change the stiffness of the structure by adjusting the DC voltage, thereby altering the resonant frequency to achieve the purpose of mode-matching. Complex algorithms are used for parameter fitting [13,14], identification [5,7], and prediction [17] to achieve real-time frequency tuning. These strategies can effectively eliminate the frequency split, but they need a large amount of original data acquisition, and the general applicability is not ideal. Some literature utilize the characteristics of the gyroscope output signal to reduce the frequency mismatch between the two operating modes. When Δf=0, the amplitude of the Coriolis signal and the quadrature signal reach the maximum [2,3,16], and the phase difference between the quadrature signal and the drive detection signal is 90${}^{\circ }$ [4,8,18]. However, these frequency tuning strategies do not work properly if the input angular velocity changed. The frequency tuning strategies that can satisfy the normal operation of the gyroscope is to introduce low-frequency oscillation signals into the sense resonator, and realize mode-matching according to the amplitude or phase characteristics of the output signals [1,6,15,19].

This paper presents a real-time automatic tuning technology for dual-mass MEMS gyroscopes. A low frequency oscillation signal (its frequency is greater than the gyroscope’s bandwidth) is introduced into quadrature stiffness correction combs, and the degree of the frequency mismatch is then judged according to the output response of the sense mode. This paper is organized as follows. Section 2 gives the structure of the gyroscope. The theory and simulation of frequency tuning are analyzed in Section 3. In Section 4, the relevant experimental results are published to testify the theoretical analysis and contrast the gyroscope’s performance. Section 5 concludes this paper with a summary.

## 2. Dual-mass MEMS Gyroscope Structure

### 2.1. Gyroscope Overall Structure

**Figure 1:**
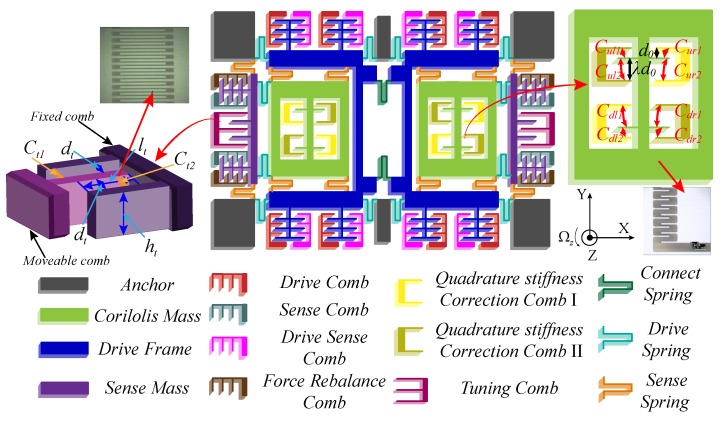
Mechanical model of dual-mass MEMS gyroscope.

In this paper, the structure of the dual-mass MEMS gyroscope is shown in Figure 1. Two fully symmetrical masses perform a simple harmonic vibration of equal amplitude and reverse phase along the *X*-axis direction under the action of electrostatic driving force. Due to the Coriolis effect, the Coriolis mass drives the sense comb to move along the *Y*-axis through the U-shaped connecting spring when the angular velocity exists in the sensitive axis (*Z*-axis), which causes the relative motion between the moveable electrode and the fixed electrode of the sense combs, resulting in the change in differential detection capacitance. By measuring the amount of capacitance change, the corresponding input angular velocity can be obtained. In addition, the gyroscope has another three combs: the sense feedback comb, the quadrature stiffness correction comb, and the frequency tuning comb. The area-changing force rebalance combs are used to suppress the movement by Coriolis force, the angular velocity information is obtained indirectly by the size of the feedback force, and the force rebalance combs will not change the resonant of the two operating modes [6]. The quadrature stiffness correction combs with unequal spacing are designed in Coriolis mass for restraining the quadrature error. The gap-changing frequency tuning combs are for applying a change in the resonant frequency of the sense mode.

The MEMS gyroscope consists of two operating modes—the drive mode and the sense mode. Both modes can be considered as a “spring-mass-damping” second-order system. According to Newton’s second law, furthermore, only the effects of quadrature coupling stiffness are considered, simplified dynamics equation for the drive mode and sense mode of MEMS gyroscope are obtained as follows:

(1) $$\left[\begin{array}{cc} m_{x} & 0\\ 0 & m_{y} \end{array}\right]\left[\begin{array}{c} \ddot{x}\\ \ddot{y} \end{array}\right]+\left[\begin{array}{cc} c_{x} & 0\\ 0 & c_{y} \end{array}\right]\left[\begin{array}{c} \dot{x}\\ \dot{y} \end{array}\right]+\left[\begin{array}{cc} k_{xx} & k_{xy}\\ k_{yx} & k_{yy} \end{array}\right]\left[\begin{array}{c} x\\ y \end{array}\right]=\left[\begin{array}{c} F_{x}\\ -2m_{c}\Omega_{z}\dot{x}+F_{y} \end{array}\right]$$

where *x* and *y* are the displacement of drive mode and sense mode, respectively; $m_{x}$ and $m_{y}$ are the equivalent mass of the two modes, respectively; $c_{x}$, $k_{xx}$ and $c_{y}$, $k_{yy}$ are the damp coefficients and the stiffness coefficients of the drive and sense modes; $k_{xy}$ and $k_{yx}$ are the coupling stiffness coefficients in each mode; $F_{x}$ and $F_{y}$ is the external force applied to drive mode and sense mode, respectively; $m_{c}$ is the Coriolis mass and $m_{c}\approx m_{y}$; $\Omega_{z}$ is the input angular velocity with respect to the Z-axis.

Assume the electrostatic driving force in the drive mode is $F_{x}=A_{F}sin\omega_{d}t$, where $A_{F}$ is the amplitude of electrostatic driving force, and $\omega_{d}$ is the frequency of electrostatic driving force. The gyroscope system uses phase-locked loop technology to track the resonant frequency of the driving mode, that is $\omega_{d}=\omega_{x}$. Substituting $F_{x}$ into Equation (1), the stationary solution of x and y can be obtained as

(2) $$x\left(t\right)=A_{x}\sin\left(\omega_{d}t+\varphi_{x}\right)$$

(3) $$y\left(t\right)=\underset{\mathrm{Coriolis}\;\mathrm{Singal}}{\underset{︸}{A_{c}\cos\left(\omega_{d}t+\varphi_{x}+\varphi_{y}\right)}}+\underset{\mathrm{Quadrature}\;\mathrm{Singal}}{\underset{︸}{A_{q}\sin\left(\omega_{d}t+\varphi_{x}+\varphi_{y}\right)}}$$

where $A_{x}$, $A_{c}$, and $A_{q}$ can be written as

(4) $$A_{x}=\frac{A_{F}/m_{x}}{\omega_{x}^{2}\sqrt{{\left(1-\frac{\omega_{d}^{2}}{\omega_{x}^{2}}\right)}^{2}+{\left(\frac{\omega_{d}}{Q_{x}\omega_{x}}\right)}^{2}}}$$

(5) $$A_{c}=\frac{2\Omega_{z}A_{x}\omega_{d}}{\omega_{y}^{2}\sqrt{{\left(1-\frac{\omega_{d}^{2}}{\omega_{y}^{2}}\right)}^{2}+{\left(\frac{\omega_{d}}{Q_{y}\omega_{y}}\right)}^{2}}}$$

(6) $$A_{q}=\frac{-\Omega_{z}A_{x}k_{xy}/m_{y}}{\omega_{y}^{2}\sqrt{{\left(1-\frac{\omega_{d}^{2}}{\omega_{y}^{2}}\right)}^{2}+{\left(\frac{\omega_{d}}{Q_{y}\omega_{y}}\right)}^{2}}}$$

(7) $$\varphi_{x}=-\arctan\frac{\omega_{x}\omega_{d}}{Q_{x}\left(\omega_{x}^{2}-\omega_{d}^{2}\right)}$$

(8) $$\varphi_{y}=-\arctan\frac{\omega_{y}\omega_{d}}{Q_{y}\left(\omega_{y}^{2}-\omega_{d}^{2}\right)}$$

where $\omega_{x}=\sqrt{k_{xx}/m_{x}}$ ($\omega_{x}=2\pi f_{x}$) and $\omega_{y}=\sqrt{k_{yy}/m_{y}}$ ($\omega_{y}=2\pi f_{y}$) are the angular frequencies of the drive mode and sense mode, respectively; $Q_{x}=\frac{\omega_{x}m_{x}}{c_{x}}$ and $Q_{y}=\frac{\omega_{y}m_{y}}{c_{y}}$ are the quality factors of each mode.

Let $\Delta \omega =|\omega_{y}-\omega_{x}|$. The mechanical sensitivity of dual-mass MEMS gyroscope can then be expressed as

(9) $$\begin{array}{cc} S & =\frac{2A_{F}Q_{x}}{m_{x}\omega_{y}^{2}}\;\cdot \;\frac{1}{\sqrt{{\left(1-\frac{\omega_{d}^{2}}{\omega_{y}^{2}}\right)}^{2}+{\left(\frac{\omega_{d}}{Q_{y}\omega_{y}}\right)}^{2}}}\\ & \approx \frac{A_{x}}{\Delta \omega }. \end{array}$$

When $\omega_{x}=\omega_{y}$, the mechanical sensitivity reaches its maximum value:

(10) $$S_{max}=\frac{2A_{F}Q_{x}Q_{y}}{m_{x}\omega_{d}^{3}}=\frac{2A_{x}Q_{y}}{\omega_{d}}.$$

**Figure 2:**
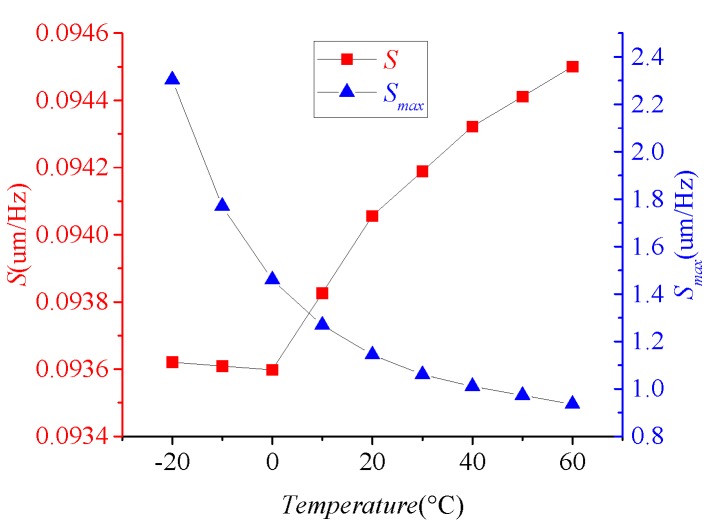
The mechanical sensitivity at different temperature (left red *y*-axis represents *S* and right blue *y*-axis represents $S_{max}$).

Based on Equations (9) and (10), the value of $\omega_{x}$, $\omega_{y}$, $Q_{x}$, and $Q_{y}$ will affect the size of *S* and $S_{max}$. Figure 2 shows the values of *S* and $S_{max}$ at different temperatures when $A_{x}=5$ um. It can be concluded that *S* does not change monotonically with temperature, but its overall trend increases with temperature and $S_{max}$ decreases with increasing temperature. Furthermore, $S_{max}$ is greater than *S* at the same temperature. When the amplification factor of the interface circuit is fixed, the scaling factor is proportional to the mechanical sensitivity.

**Figure 3:**
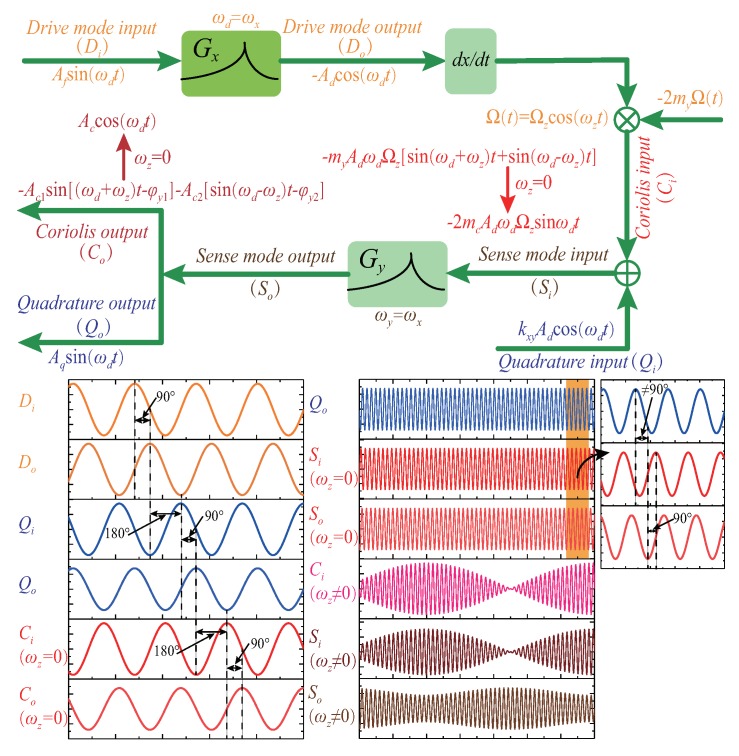
The phase relationship between the drive mode and sense mode signals.

Figure 3 shows the phase relationship between the drive mode and sense mode signals and takes into account changes in the input angular velocity. When the input angular velocity exists or changes, the technologies in [2,3,4,8,16,18] can not effectively identify the Coriolis signal and the quadrature signal, which leads to frequency tuning failure.

### 2.2. Quadrature Stiffness Correction Structure

Quadrature stiffness correction structure as shown in Figure 1, these combs have the two degrees of freedom in both *X* and *Y* directions, which is located in the Coriolis mass. In addition, they are arranged at unequal intervals, with unequal pitch ratio λ>1. The correction voltages $V_{q1}$ and $V_{q2}$ are respectively applied to Quadrature Electrodes 1 and 2. The right mass is used as an example to analyze the working mechanism of the quadrature stiffness correction combs. The stiffness matrix of the correction combs under electrostatic force can be expressed as

(11) $$\left[\begin{array}{cc} k_{qxx} & k_{qxy}\\ k_{qyx} & k_{qyy} \end{array}\right]=\left[\begin{array}{cc} -\frac{\partial F_{qx}}{\partial x} & -\frac{\partial F_{qx}}{\partial y}\\ -\frac{\partial F_{qy}}{\partial x} & -\frac{\partial F_{qy}}{\partial y} \end{array}\right]$$

where $F_{qx}$ and $F_{qy}$ can be written as

(12) $$F_{qx}=\frac{1}{2}V_{q1}^{2}\left(\frac{\partial C_{ul1}}{\partial x}+\frac{\partial C_{ul2}}{\partial x}+\frac{\partial C_{dr1}}{\partial x}+\frac{\partial C_{dr2}}{\partial x}\right)+\frac{1}{2}V_{q2}^{2}\left(\frac{\partial C_{dl1}}{\partial x}+\frac{\partial C_{dl2}}{\partial x}+\frac{\partial C_{ur1}}{\partial x}+\frac{\partial C_{ur2}}{\partial x}\right)$$

(13) $$F_{qy}=\frac{1}{2}V_{q1}^{2}\left(\frac{\partial C_{ul1}}{\partial y}+\frac{\partial C_{ul2}}{\partial y}+\frac{\partial C_{dr1}}{\partial y}+\frac{\partial C_{dr2}}{\partial y}\right)+\frac{1}{2}V_{q2}^{2}\left(\frac{\partial C_{dl1}}{\partial y}+\frac{\partial C_{dl2}}{\partial y}+\frac{\partial C_{ur1}}{\partial y}+\frac{\partial C_{ur2}}{\partial y}\right)$$

where $F_{qx}$ and $F_{qy}$ are the electrostatic force generated by the quadrature correction combs in the *X*- and *Y*-axes, respectively.

Consider the displacement of the sense mode $y\ll d_{0}$. Moreover, $V_{q1}=V_{d}+V_{q}$ and $V_{q2}=V_{d}-V_{q}$. Thus, Equation (11) can be simplified as

(14) $$\left[\begin{array}{cc} k_{qxx} & k_{qxy}\\ k_{qyx} & k_{qyy} \end{array}\right]=\left[\begin{array}{cc} 0 & -\frac{4n_{q}\varepsilon_{0}h_{q}}{d_{q}^{2}}\left(1-\frac{1}{\lambda^{2}}\right)V_{d}V_{q}\\ -\frac{4n_{q}\varepsilon_{0}h_{q}}{d_{q}^{2}}\left(1-\frac{1}{\lambda^{2}}\right)V_{d}V_{q} & -\frac{4n_{q}\varepsilon_{0}h_{q}l_{q}}{d_{q}^{3}}\left(1+\frac{1}{\lambda^{3}}\right)\left(V_{d}^{2}+V_{q}^{2}\right) \end{array}\right]$$

where $\varepsilon_{0}$ is the vacuum permittivity; $h_{q}$ is the thickness of the correct comb; $l_{q}$ is the initial combing length of fixed comb and movable comb; $d_{q}$ is the distance between the fixed comb and moveable comb; $n_{q}$ is the number of quadrature stiffness correction combs; $V_{d}$ is the preset fixed DC benchmark voltage; $V_{q}$ is the quadrature adjustment voltage.

**Figure 4:**
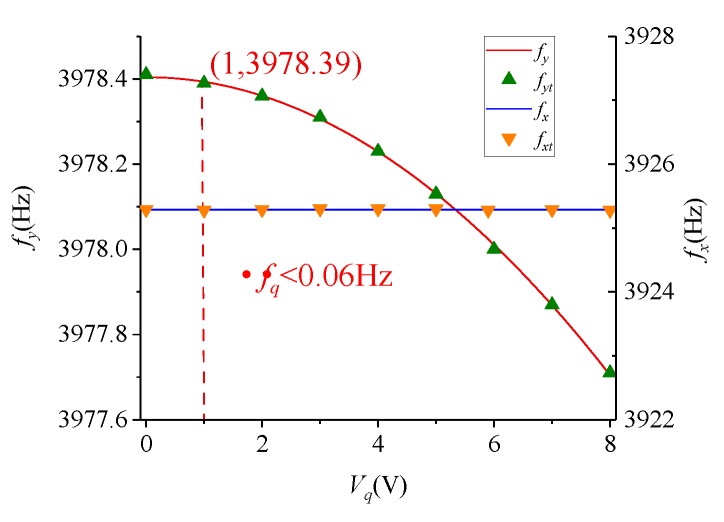
The effect of $V_{q}$ on $f_{x}$ and $f_{y}$.

From Equation (14), the quadrature stiffness correction structure does not affect the stiffness of drive mode. In the drive mode and the sense mode, it can produce a negative stiffness to counteract the quadrature coupling stiffness. In addition, $F_{qy}$ will generate the negative stiffness in the sense mode, and affected the resonance frequency of sense mode. Figure 4 illustrated the effect of $V_{q}$ on $f_{x}$ and $f_{y}$. The continuous curves are the theoretical calculation data, and the discrete points are the measured data. When $V_{d}=2.048$ V and $V_{q}=1$ V, both the test value and calculated value indicate the effect of $V_{q}$ on $f_{y}$ is less than 0.06 Hz. This makes it feasible to use the quadrature stiffness correction structure to generate modulation signals for obtaining frequency mismatch information.

### 2.3. Frequency Tuning Structure

The frequency tuning combs are also shown in Figure 1. The combs are the gap-changing structure and with an equal gap. The movable combs can only move in the *Y* direction. Taking a single frequency tuning comb as an example, the frequency adjustment voltage $V_{t}$ is applied to the fixed comb. When the movable comb moves along the *Y*-axis, the capacitance variation on both sides of the fixed comb can be described as

(15) $$\left\{\begin{array}{c} C_{t1}=\frac{\varepsilon_{0}h_{t}l_{t}}{d_{t}-y}\\ C_{t1}=\frac{\varepsilon_{0}h_{t}l_{t}}{d_{t}+y} \end{array}\right.$$

where $C_{t1}$ and $C_{t2}$ are the capacitance between the fixed comb and the movable comb; $h_{t}$ is the thickness of the comb; $l_{t}$ is the initial combing length of the fixed comb and the movable comb; $d_{t}$ is the distance between the fixed comb and the moveable comb.

Furthermore, the stiffness matrix of the frequency tuning comb under electrostatic force can be expressed as

(16) $$\left[\begin{array}{cc} k_{txx} & k_{txy}\\ k_{tyx} & k_{tyy} \end{array}\right]=\left[\begin{array}{cc} -\frac{\partial F_{tx}}{\partial x} & -\frac{\partial F_{tx}}{\partial y}\\ -\frac{\partial F_{ty}}{\partial x} & -\frac{\partial F_{ty}}{\partial y} \end{array}\right]$$

where $F_{tx}$ and $F_{ty}$ can be written as

(17) $$F_{tx}=0,$$

(18) $$F_{ty}=F_{t1y}+F_{t2y}=\frac{1}{2}V_{t}^{2}\left(\frac{\partial C_{t1}}{\partial y}+\frac{\partial C_{t2}}{\partial y}\right)=\frac{\varepsilon_{0}h_{t}l_{t}V_{t}^{2}}{2}\left[\frac{1}{{\left(d_{t}-y\right)}^{2}}-\frac{1}{{\left(d_{t}+y\right)}^{2}}\right]$$

where $F_{tx}$ and $F_{ty}$ is the electrostatic force generated by the two capacitors in the X and Y directions, respectively.

Consider $y\ll d_{t}$, Equation (16) can be simplified as

(19) $$\left[\begin{array}{cc} k_{txx} & k_{txy}\\ k_{tyx} & k_{tyy} \end{array}\right]=\left[\begin{array}{cc} 0 & 0\\ 0 & -\frac{\varepsilon_{0}h_{t}l_{t}}{d_{t}^{3}}V_{t}^{2} \end{array}\right].$$

**Figure 5:**
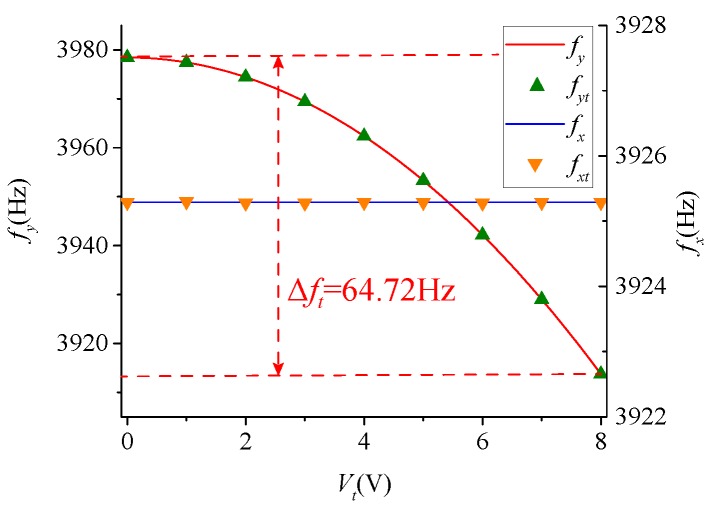
The effect of $V_{t}$ on $f_{x}$ and $f_{y}$.

According to Equation (19), $V_{t}$ does not cause a change in $\omega_{x}$. $\omega_{y}$ will monotonically decrease as $V_{t}$ increases, so that $\omega_{x}=\omega_{y}$ at a certain voltage value. Figure 5 shows the variation of $f_{x}$ and $f_{y}$ at different $V_{t}$ values. Similarly, the continuous curves are the theoretical calculation data, and the discrete points are the measured data. Moreover, the frequency adjustment capability of the 8 V DC voltage is 64.72 Hz.

## 3. Automatic Frequency Tuning System

### 3.1. Frequency Tuning Theory

Combined Equations (14) and (19), the equivalent stiffness of the sense mode can be expressed as

(20) $$k_{eyy}=k_{yy}+k_{tyy}+k_{qyy}.$$

The resonant frequency of the sense mode under $V_{t}$, $V_{d}$, and $V_{q}$ can be represented as

(21) $$\omega_{eyy}=\sqrt{\frac{k_{yy}}{m_{y}}-\frac{n_{t}\varepsilon_{0}h_{t}l_{t}}{m_{y}d_{t}^{3}}V_{t}^{2}-\frac{4n_{q}\varepsilon_{0}h_{q}l_{q}}{m_{y}d_{q}^{3}}\left(1+\frac{1}{\lambda^{3}}\right)\left(V_{d}^{2}+V_{q}^{2}\right)}.$$

**Figure 6:**
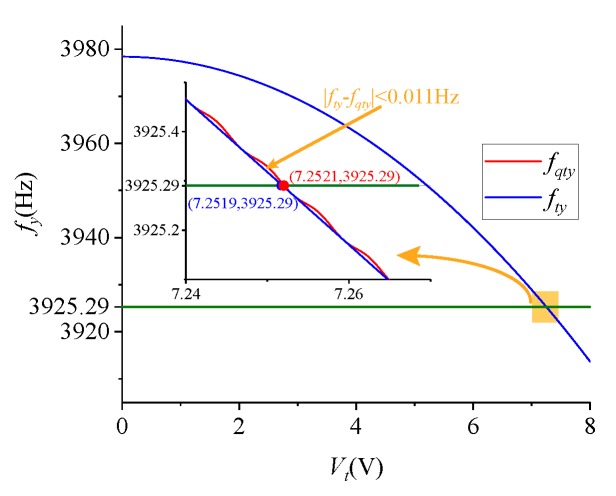
The effect of $V_{t}$ and $V_{q}$ on $f_{y}$.

In this paper, $V_{d}=2.048$ V. The influence of $V_{t}$ and $V_{q}$ on $f_{y}$ is shown in Figure 6. $f_{qty}$ and $f_{ty}$ respectively represent the variation curve of $f_{y}$ when $V_{q}$ is a sine wave ($V_{q}=1\times \sin\left(160\pi t\right)$ V) and a direct current amount ($V_{q}=1$ V). When $V_{q}=1\times \sin\left(\omega_{t}t\right)$ V, the resonant frequency of the sense mode will produce sinusoidal fluctuations, but the maximum frequency difference between $f_{qty}$ and $f_{ty}$ is less than 0.011 Hz. Moreover, according to Equation (19) and Figure 5, the voltage of 0.2 mV only causes a deviation of $4\times 10^{-8}$ Hz. Therefore, the effect of the sinusoidal signal applied to the quadrature stiffness combs on $f_{y}$ can be neglected, which demonstrates the feasibility of frequency tuning in the following section.

**Figure 7:**
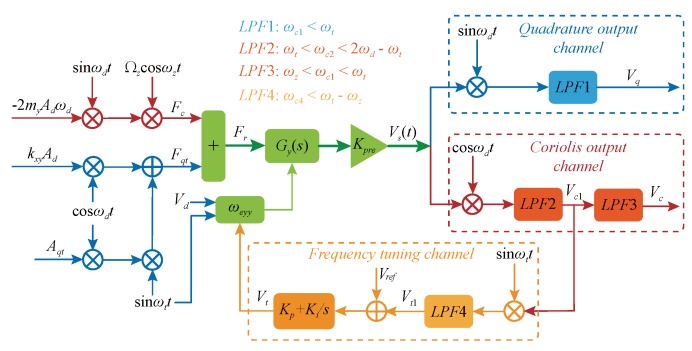
Schematic control loop for the automatic frequency tuning system.

The automatic frequency tuning loop is shown in Figure 7. The quadrature stiffness correction combs are applied with the low frequency sinusoidal voltage ($V_{q}$) and DC benchmark voltage ($V_{d}$), which can equivalently produce a modulation excitation signal including $\omega_{t}$ and $\omega_{x}$. By identifying the output response of the gyroscope under the excitation signal, the frequency matching degree of the two operating modes can be distinguished. The Coriolis signal, the quadrature signal, and the frequency mismatch signal can be obtained by different multiplication demodulations. Figure 7 also shows the configuration of the cut-off frequency for the four low-pass filters. In the frequency tuning loop, a proportional integral controller is used to adjust the frequency tuning voltage to change the resonance frequency of the sense mode.

The alternating voltage applied to the quadrature stiffness correction combs can be expressed as

(22) $$V_{q}=A_{t}\sin\omega_{t}t$$

where $A_{t}$ and $\omega_{t}$ are the amplitude and frequency of the low frequency sinusoidal signal, respectively.

Combined Equations (14) and (22), the equivalent input force generated by the quadrature channel can be expressed as

(23) $$\begin{array}{cc} F_{qt} & =A_{d}\cos\omega_{d}t*\left[k_{xy}-\frac{4n_{q}\varepsilon_{0}h_{q}}{d_{0}^{2}}\left(1-\frac{1}{\lambda^{2}}\right)V_{d}A_{t}\sin\omega_{t}t\right]\\ & =k_{xy}A_{d}\cos\omega_{d}t+A_{qt}\cos\omega_{d}t*\sin\omega_{t}t. \end{array}$$

Considering the variation of $\Omega_{z}$, the input resultant force of the sense mode can be given as

(24) $$\begin{array}{cc} F_{r} & =F_{c}+F_{qt}\\ & =\underset{\mathrm{Coriolis}\;\mathrm{force}}{\underset{︸}{-2m_{y}A_{d}\omega_{d}\Omega_{z}\cos\omega_{z}t*\sin\omega_{d}t}}+\underset{\mathrm{Quadrature}\;\mathrm{coupling}\;\mathrm{force}}{\underset{︸}{k_{xy}A_{d}\cos\omega_{d}t}}+\underset{\mathrm{Quadrature}\;\mathrm{modulation}\;\mathrm{force}}{\underset{︸}{A_{qt}\cos\omega_{d}t*\sin\omega_{t}t}}. \end{array}$$

The output of the sense mode should be the sum of the responses of the above three forces,

(25) $$\begin{array}{cc} V_{s} & =V_{c}+V_{qt}\\ & =\underset{\mathrm{Coriolis}\;\mathrm{signal}}{\underset{︸}{A_{c1}\sin\left[\left(\omega_{d}+\omega_{z}\right)t+\varphi_{\omega_{d}+\omega_{z}}\right]-A_{c2}\sin\left[\left(\omega_{d}-\omega_{z}\right)t+\varphi_{\omega_{d}-\omega_{z}}\right]}}\\ & \;+\underset{\mathrm{Frequency}\;\mathrm{tuning}\;\mathrm{signal}}{\underset{︸}{A_{t1}\sin\left[\left(\omega_{d}+\omega_{t}\right)t+\varphi_{\omega_{d}+\omega_{t}}\right]-A_{t2}\sin\left[\left(\omega_{d}-\omega_{t}\right)t+\varphi_{\omega_{d}-\omega_{t}}\right]}}\\ & \;+\underset{\mathrm{Quadrature}\;\mathrm{signal}}{\underset{︸}{A_{q}\cos\left(\omega_{d}+\varphi_{q}\right)}} \end{array}$$

where

(26) $$A_{c1}=\frac{-A_{d}\omega_{d}\Omega_{z}K_{pre}}{\sqrt{{\left[\omega_{y}^{2}-{\left(\omega_{d}+\omega_{z}\right)}^{2}\right]}^{2}+{\left[\omega_{y}\left(\omega_{d}+\omega_{z}\right)/Q_{y}\right]}^{2}}}$$

(27) $$A_{c2}=\frac{-A_{d}\omega_{d}\Omega_{z}K_{pre}}{\sqrt{{\left[\omega_{y}^{2}-{\left(\omega_{d}-\omega_{z}\right)}^{2}\right]}^{2}+{\left[\omega_{y}\left(\omega_{d}-\omega_{z}\right)/Q_{y}\right]}^{2}}}$$

(28) $$A_{t1}=\frac{A_{qt}K_{pre}/2m_{y}}{\sqrt{{\left[\omega_{y}^{2}-{\left(\omega_{d}+\omega_{t}\right)}^{2}\right]}^{2}+{\left[\omega_{y}\left(\omega_{d}+\omega_{t}\right)/Q_{y}\right]}^{2}}}$$

(29) $$A_{t2}=\frac{A_{qt}K_{pre}/2m_{y}}{\sqrt{{\left[\omega_{y}^{2}-{\left(\omega_{d}-\omega_{t}\right)}^{2}\right]}^{2}+{\left[\omega_{y}\left(\omega_{d}-\omega_{t}\right)/Q_{y}\right]}^{2}}}$$

(30) $$A_{q}=\frac{k_{xy}A_{d}/m_{y}}{\sqrt{{\left(\omega_{y}^{2}-\omega_{d}^{2}\right)}^{2}+{\left(\omega_{y}\omega_{d}/Q_{y}\right)}^{2}}}$$

(31) $$\varphi_{\omega_{d}+\omega_{z}}=-\arctan\frac{\omega_{y}\left(\omega_{d}+\omega_{z}\right)}{Q_{y}\left[\omega_{y}^{2}-{\left(\omega_{d}+\omega_{z}\right)}^{2}\right]}$$

(32) $$\varphi_{\omega_{d}-\omega_{z}}=-\arctan\frac{\omega_{y}\left(\omega_{d}-\omega_{z}\right)}{Q_{y}\left[\omega_{y}^{2}-{\left(\omega_{d}-\omega_{z}\right)}^{2}\right]}$$

(33) $$\varphi_{\omega_{d}+\omega_{t}}=-\arctan\frac{\omega_{y}\left(\omega_{d}+\omega_{t}\right)}{Q_{y}\left[\omega_{y}^{2}-{\left(\omega_{d}+\omega_{t}\right)}^{2}\right]}$$

(34) $$\varphi_{\omega_{d}-\omega_{t}}=-\arctan\frac{\omega_{y}\left(\omega_{d}-\omega_{t}\right)}{Q_{y}\left[\omega_{y}^{2}-{\left(\omega_{d}-\omega_{t}\right)}^{2}\right]}$$

(35) $$\varphi_{q}=-\arctan\frac{\omega_{y}\omega_{d}}{Q_{y}\left(\omega_{y}^{2}-\omega_{d}^{2}\right)}.$$

Therefore, the output of low pass filter LPF2 can be obtained as

(36) $$\begin{array}{cc} V_{c1} & =\frac{1}{2}A_{c1}\sin\left(\omega_{z}t+\varphi_{\omega_{d}+\omega_{z}}\right)-\frac{1}{2}A_{c2}\sin\left(\omega_{z}t-\varphi_{\omega_{d}-\omega_{z}}\right)\\ & \;+\frac{1}{2}A_{t1}\sin\left(\omega_{t}t+\varphi_{\omega_{d}+\omega_{t}}\right)+\frac{1}{2}A_{t2}\sin\left(\omega_{t}t-\varphi_{\omega_{d}-\omega_{t}}\right)\\ & \;+\frac{1}{2}A_{q}\cos\varphi_{q}. \end{array}$$

When $\omega_{x}=\omega_{y}$, the value of $\cos\varphi_{q}$ is zero. The signal $V_{c1}$ pass through the low pass filter LPF3, the Coriolis output can be written as

(37) $$V_{c}=\frac{1}{2}A_{c1}\sin\left(\omega_{z}t+\varphi_{\omega_{d}+\omega_{z}}\right)-\frac{1}{2}A_{c2}\sin\left(\omega_{z}t-\varphi_{\omega_{d}-\omega_{z}}\right).$$

According to Equation (37), the Coriolis output can eliminate the interference from the quadrature signal and low frequency input signal, and correctly reflect the input angular velocity.

Similarly, $V_{q}$ and $V_{t1}$ can be expressed as

(38) $$V_{q}=\frac{1}{2}A_{c1}\cos\left(\omega_{z}t+\varphi_{\omega_{d}+\omega_{z}}\right)-\frac{1}{2}A_{c2}\cos\left(\omega_{z}t-\varphi_{\omega_{d}-\omega_{z}}\right)-\frac{1}{2}A_{q}\sin\varphi_{q}$$

(39) $$\begin{array}{cc} V_{t1} & =\frac{1}{4}A_{t1}\cos\varphi_{\omega_{d}+\omega_{t}}+\frac{1}{4}A_{t2}\cos\varphi_{\omega_{d}-\omega_{t}}\\ & =\frac{A_{qt}K_{pre}}{8m_{y}}\left(t_{eq1}+t_{eq2}\right) \end{array}$$

where $t_{eq1}$ and $t_{eq2}$ can be given as

(40) $$t_{eq1}=\frac{\omega_{y}^{2}-{\left(\omega_{d}+\omega_{t}\right)}^{2}}{{\left[\omega_{y}^{2}-{\left(\omega_{d}+\omega_{t}\right)}^{2}\right]}^{2}+{\left[\omega_{y}\left(\omega_{d}+\omega_{t}\right)/Q_{y}\right]}^{2}}$$

(41) $$t_{eq2}=\frac{\omega_{y}^{2}-{\left(\omega_{d}-\omega_{t}\right)}^{2}}{{\left[\omega_{y}^{2}-{\left(\omega_{d}-\omega_{t}\right)}^{2}\right]}^{2}+{\left[\omega_{y}\left(\omega_{d}-\omega_{t}\right)/Q_{y}\right]}^{2}}.$$

According to Equation (38), the quadrature output contains the momentum associated with $\omega_{z}$. Since the quadrature quantity is a slow variable, in the actual system, the interference can be filtered out as much as possible by a low pass filter (*LPF*1) with a very low cut-off frequency.

$\omega_{y}=\omega_{d}+\Delta \omega$ is substituted into Equation (39), and the expression for Δω is as follows:

(42) $$V\left(\Delta \omega \right)=\frac{A_{qt}K_{pre}}{8m_{y}}\left(V_{\Delta \omega 1}+V_{\Delta \omega 2}\right)$$

where $V_{\Delta \omega 1}$ and $V_{\Delta \omega 2}$ can be obtained as

(43) $$V_{\Delta \omega 1}=\frac{{\left(\omega_{d}+\Delta \omega \right)}^{2}-{\left(\omega_{d}+\omega_{t}\right)}^{2}}{{\left[{\left(\omega_{d}+\Delta \omega \right)}^{2}-{\left(\omega_{d}+\omega_{t}\right)}^{2}\right]}^{2}+{\left[\left(\omega_{d}+\Delta \omega \right)\left(\omega_{d}+\omega_{t}\right)/Q_{y}\right]}^{2}}$$

(44) $$V_{\Delta \omega 2}=\frac{{\left(\omega_{d}+\Delta \omega \right)}^{2}-{\left(\omega_{d}-\omega_{t}\right)}^{2}}{{\left[{\left(\omega_{d}+\Delta \omega \right)}^{2}-{\left(\omega_{d}-\omega_{t}\right)}^{2}\right]}^{2}+{\left[\left(\omega_{d}+\Delta \omega \right)\left(\omega_{d}-\omega_{t}\right)/Q_{y}\right]}^{2}}.$$

Let Δω=0 in Equation (42). The reference voltage for frequency tuning can be obtained as follows:

(45) $$V_{ref}=V\left(0\right)=\frac{A_{qt}K_{pre}}{8m_{y}}\left(V_{ref1}+V_{ref2}\right)$$

where $V_{ref1}$ and $V_{ref2}$ can be written as

(46) $$V_{ref1}=\frac{\omega_{d}^{2}-{\left(\omega_{d}+\omega_{t}\right)}^{2}}{{\left[\omega_{d}^{2}-{\left(\omega_{d}+\omega_{t}\right)}^{2}\right]}^{2}+{\left[\omega_{d}\left(\omega_{d}+\omega_{t}\right)/Q_{y}\right]}^{2}}$$

(47) $$V_{ref2}=\frac{\omega_{d}^{2}-{\left(\omega_{d}-\omega_{t}\right)}^{2}}{{\left[\omega_{d}^{2}-{\left(\omega_{d}-\omega_{t}\right)}^{2}\right]}^{2}+{\left[\omega_{d}\left(\omega_{d}-\omega_{t}\right)/Q_{y}\right]}^{2}}.$$

**Figure 8:**
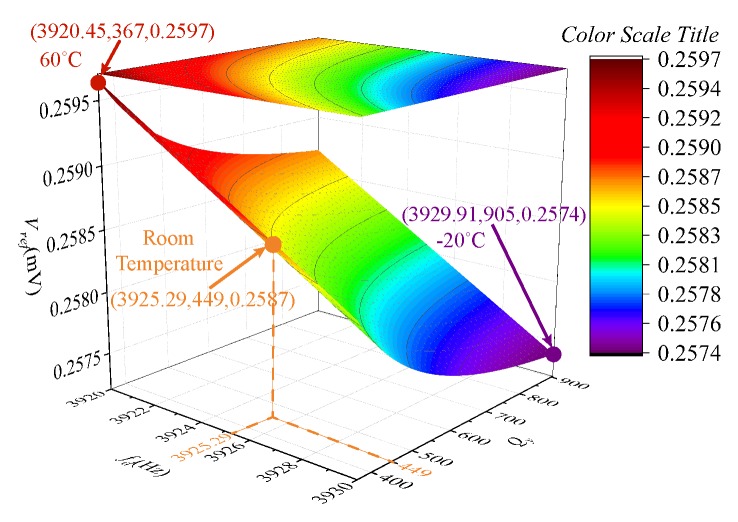
The effect of $\omega_{d}$ and $Q_{y}$ on $V_{ref}$.

After the gyroscope structure and circuit parameters are determined, each parameter in Equation (45) is a known quantity except for $\omega_{d}$ and $Q_{y}$. $\omega_{d}$ can be acquired by the drive mode control system [20], and $Q_{y}$ is considered a function of the change with $\omega_{d}$ [21]. Figure 8 shows the relationship between $\omega_{d}$, $Q_{y}$, and $V_{ref}$. The $V_{ref}$ varies from 0.2597 to 0.2574 mV over a wide temperature range (−20 to 60 ${}^{\circ }$C).

### 3.2. Frequency Tuning System Analysis

According to the principle as shown in Figure 7, an automatic frequency tuning system based on low frequency modulation excitation was built on the Simulink simulation platform. The simulation parameters are listed in Table 1. The quadrature stiffness correction comb was originally designed to suppress the quadrature coupling stiffness by applying a slowly varying DC voltage, so the comb cannot respond to a high frequency input. In order to balance the response frequency and the gyroscope’s bandwidth, $\omega_{t}=80$ Hz is selected. In addition, since the force rebalance comb does not change the $f_{x}$ and $f_{y}$ [6], the open-loop and closed-loop detection methods do not interfere with the operation of the frequency tuning system, so the open-loop detection method is adopted in the simulation analysis.

**Figure 9:**
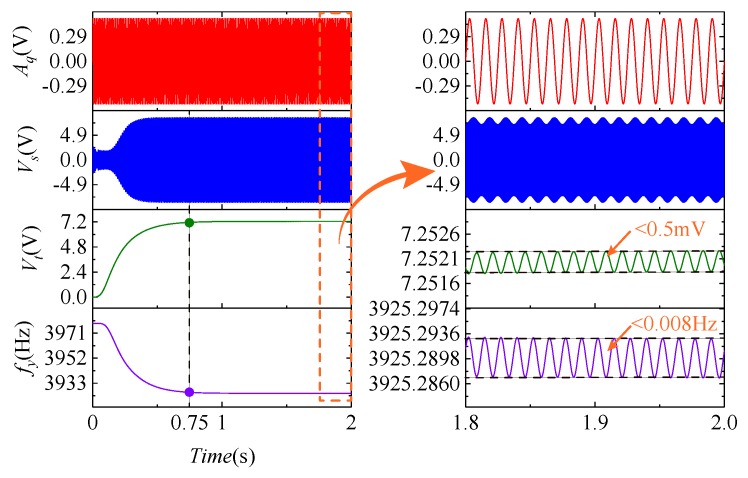
The curves of observation points in the frequency tuning system.

Figure 9 shows the curves of some observation points when the frequency tuning system is working normally ($\Omega_{z}=50^{\circ }$/s, $\Omega_{q}=100^{\circ }$/s). The first curve is the low frequency input signal ($\omega_{t}=80$ Hz) that applied to the quadrature stiffness correction combs, and its function is to cause the gyroscope to generate a modulated signal containing frequency split information. The second curve is the gyroscope output signal, which is characterized by the modulated signals of $\omega_{t}$ and $\omega_{x}$. The third and fourth curves represent the changes in the frequency tuning input voltage and the sense mode resonant frequency, respectively. The curves indicate that the system is in a stable state after 0.75 s, the output fluctuation of $V_{t}$ is less than 0.5 mV, and the fluctuation of the corresponding $f_{y}$ is less than 0.008 Hz.

**Figure 10:**
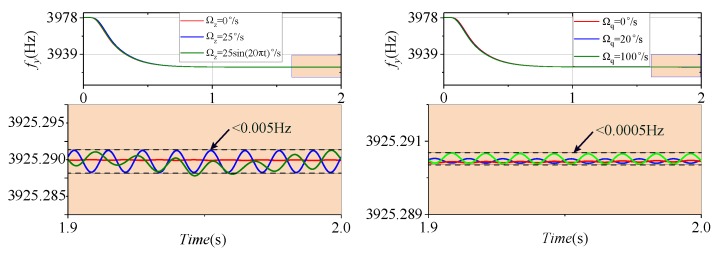
The disturbance of different $\Omega_{z}$ (**left**) and $\Omega_{q}$ (**right**) to $f_{y}$.

The effects of different $\Omega_{z}$ and $\Omega_{q}$ values (quadrature equivalent input angular velocity [22]) on $f_{y}$ are shown in Figure 10. The interference fluctuations of $\Omega_{z}$ and $\Omega_{q}$ to $f_{y}$ are less than 0.005 Hz and 0.0005 Hz, respectively. This illustrates that, when $\Omega_{z}$ and $\Omega_{q}$ exist, the frequency tuning system can still work properly, and eventually can be stabilized at the desired frequency.

**Figure 11:**
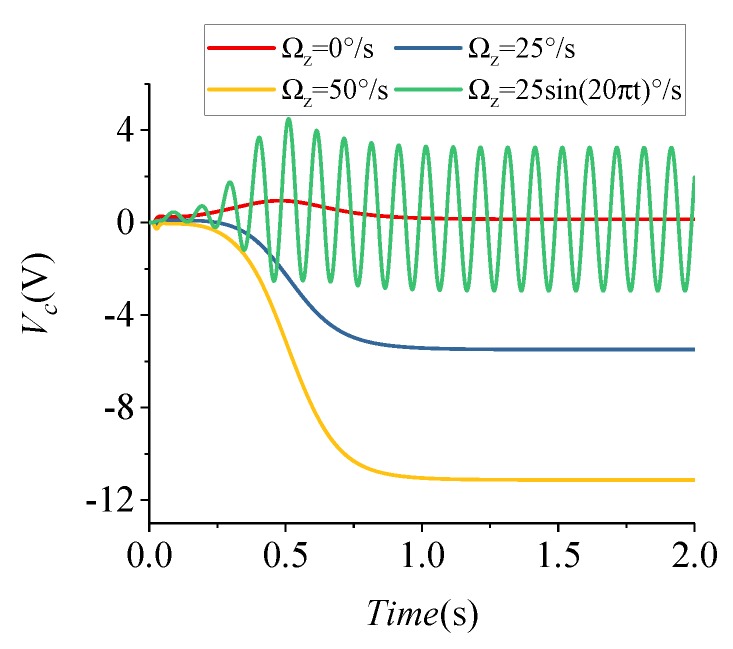
Coriolis path output curves.

Consider $\Omega_{q}$ = 100${}^{\circ }$/s, set $\Omega_{c}$ to 0${}^{\circ }$/s, $25^{\circ }/$s, 50${}^{\circ }$/s, and 25$\times \sin{\left(20\pi t\right)}^{\circ }$/s, and obtain the angular velocity output response curves, as illustrated in Figure 11. This indicates that, when the frequency tuning loop works normally, the system can still detect the input angular velocity.

## 4. Experiments

**Figure 12:**
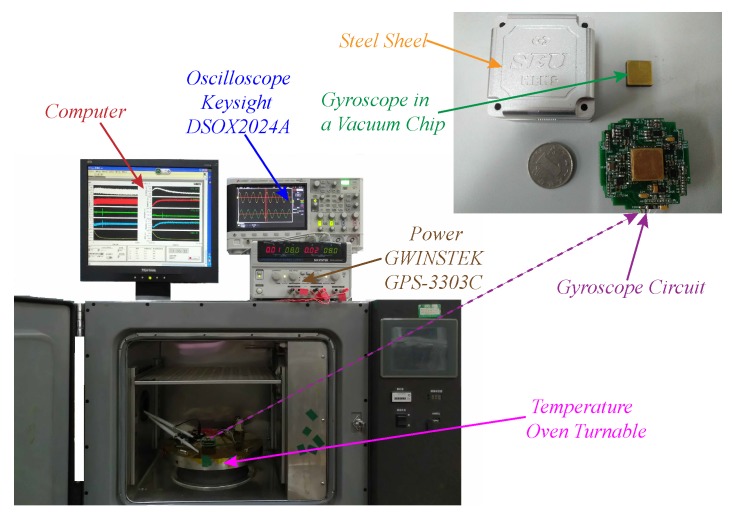
Photos of the MEMS gyroscope circuit and test equipment.

In order to verify the effectiveness of automatic frequency tuning technology in the MEMS gyroscope, the gyroscope control circuit was designed and relevant tests were conducted. Figure 12 shows the experimental test equipment and the gyroscope system circuit. The circuit is mounted on the printed circuit boards (PCBs) and its electrical signals and mechanical structure are connected to each other through metal pins. First, the PCBs are wrapped in rubber pads that protect PCBs and fabric chips from impact and vibration. The test equipment includes the power (GWINSTEK GPS-3303C, GWINSTEK, New Taipei, China) providing ±8 V DC voltage and GND, the oscilloscope (Keysight DSOX2024A, Keysight, Santa Rosa, CA, USA), which is applied to observe the different input and output signals of the gyroscope, the computer, which is devoted to measuring the gyroscope data in a variety of working conditions, and the temperature oven, providing a wide-temperature range environment and a turntable test of the bandwidth of the gyroscope.The experiments were divided into three methods: open-loop detection without frequency tuning (Test 1), open-loop detection with frequency tuning (Test 2), and closed-loop detection with frequency tuning (Test 3).

**Figure 13:**
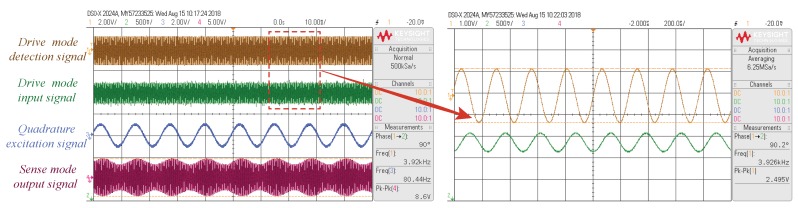
The test curves of the frequency tuning system.

The test curves of the frequency tuning system at room temperature are shown in Figure 13. These four curves are the drive mode detection signal, the drive mode input signal, the quadrature excitation signal ($V_{q}$), and the sense mode output signal. The frequency of the drive mode input signal and the drive mode detection signal were both $\omega_{x}$, and a phase difference of 90${}^{\circ }$ was maintained, while the amplitude of the drive mode detection signal remained stable. These curves indicated that the drive mode of the gyroscope was working properly. The frequency of the tuning input signal was 80 Hz, and the sense mode output signal was the modulation signal of 80 Hz and $\omega_{x}$.

**Figure 14:**
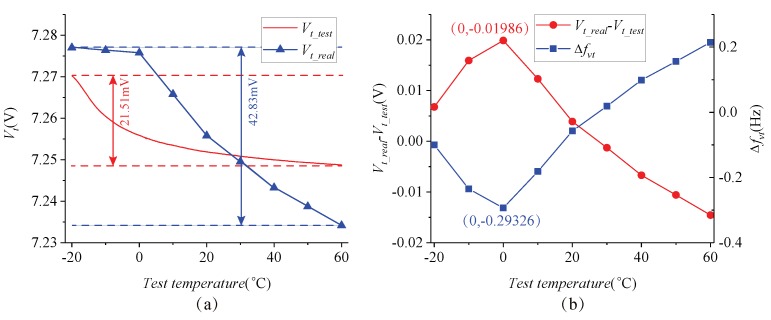
Wide temperature range test curves: (**a**) The variation of frequency tuning voltage, (**b**) The variations of $V_{t\_real}-V_{t\_test}$ and $\Delta f_{\nu t}$.

The gyroscope control circuit was placed in the temperature oven for a wide temperature range test, and the temperature range was set from -20${}^{\circ }C$–60${}^{\circ }C$. The curve of frequency tuning voltage varying with temperature was obtained under the automatic frequency tuning technology, as shown in Figure 14. $V_{t\_test}$ and $V_{t\_real}$ are the frequency tuning voltages of the temperature test and the actual temperature conditions, respectively. Among them, $V_{t\_real}$ is the frequency tuning voltage at each stable temperature condition under manual adjustment mode, which can be characterized as the real frequency tuning voltage. $\Delta f_{\nu t}$ is the frequency split in the wide temperature range test, which represents the mismatch frequency of the gyroscope adopting the frequency tuning technology. In the wide temperature range, the frequency tuning voltage was changed from 7.27021 to 7.24871 V, the maximum difference between the test tuning voltage and the real tuning voltage was 1.986 mV, and the corresponding frequency difference was 0.29326 Hz.

**Figure 15:**
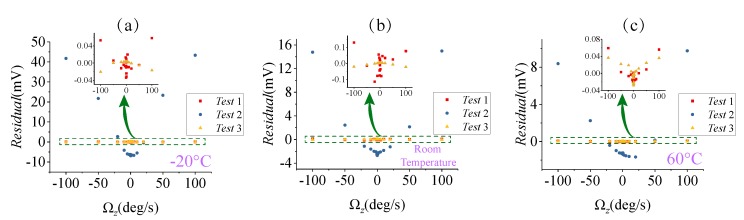
The residual errors of the scale factor under different temperatures: (**a**) −20 ${}^{\circ }$C condition, (**b**) Room temperature condition, (**c**) 60 ${}^{\circ }$C condition.

The scale factor tests (listed in Table 2) at the three different temperatures were arranged with input angular rates $\Omega_{z}$ of ±0.1${}^{\circ }$/s, ±0.2${}^{\circ }$/s, ±0.5${}^{\circ }$/s, ±1${}^{\circ }$/s, ±2${}^{\circ }$/s, ±5${}^{\circ }$/s, ±10${}^{\circ }$/s, ±20${}^{\circ }$/s, ±50${}^{\circ }$/s, and ±100${}^{\circ }$/s, and Figure 15 shows the residuals of the fit. According to Equation (10), $Q_{x}$ and $Q_{y}$ change with temperature, resulting in a large change in the scale factor value of Test 2. The scale factor of Test 2 was greater than that of Test 1, which is theoretically demonstrated in Figure 2, but its scale factor nonlinearly was degraded. When the input angular velocity is $\pm 100^{\circ }/$s, the residual error of Test 1 and Test 2 between the measured data and fitting data reached the maximum. Test 3 adopted a close-loop detection method that made the scale factor independent of the mechanical sensitivity, and the nonlinearity was improved.

The static output performance of the three tests at three different temperature was also tested, as listed in Table 3. The angle random walk (ARW) of the same test mode remained stable under different temperature conditions. However, as the temperature rose, the damping coupling increased, resulting in deterioration of the zero bias stability. Comparing Test 1 and Test 2, the static performance of Test 2 was improved due to the adoption of frequency tuning technology. The performance of Test 3 and Test 2 was basically the same.

**Figure 16:**
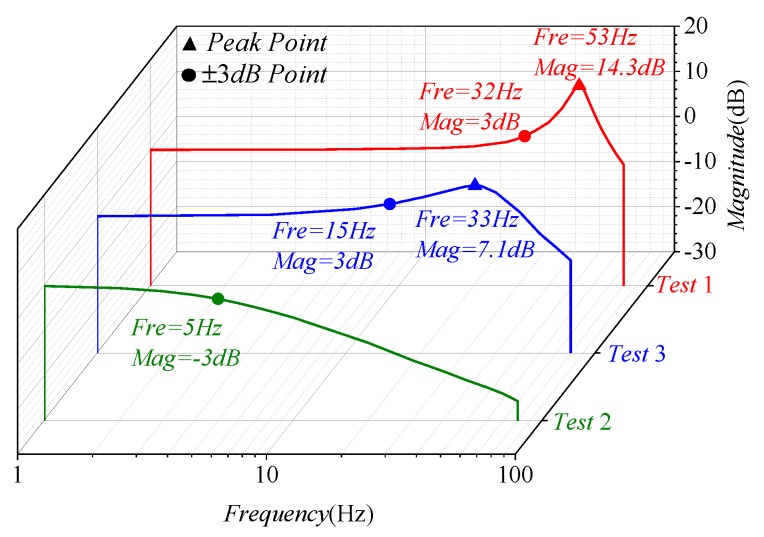
Gyroscope bandwidth under the three test methods.

The bandwidth of the gyroscope at room temperature is shown in Figure 16. The bandwidth of Test 1 was 31 Hz, which is approximately equal to 0.54Δf [20], while the detection transfer function of Test 2 can be approximated as a low-pass filter with a cut-off frequency of $\omega_{y}/2Q_{y}$ [17], so the bandwidth of Test 2 was 5 Hz. Because of the previous bandwidth expansion technology [23], the bandwidth of Test 3 reached 15 Hz.

## 5. Conclusions

This paper focuses on the automatic frequency tuning technology based on a quadrature modulation signal. The quadrature stiffness correction combs are applied to a DC benchmark voltage and a low frequency sinusoidal signal whose frequency is higher than the gyroscope’s bandwidth, which can equivalently produce a modulation excitation signal acting on the input of gyroscope. By identifying the output response of the gyroscope under this excitation signal, the frequency mismatch degree of the two operating modes can be distinguished. In order to obtain a frequency tuning signal, a Coriolis signal, and a quadrature signal, a low pass filter with different cut-off frequencies were configured for demodulation. Simulation analysis and experimental results demonstrate the feasibility of the automatic frequency tuning system. The wide temperature range test demonstrates the reliability of the frequency tuning system with a maximum mismatch frequency of less than 0.3 Hz in the range of -20${}^{\circ }C$–60${}^{\circ }C$. The scale factor test and static test of the gyroscope at three different temperatures (−20 ${}^{\circ }$C, room temperature, 60 ${}^{\circ }$C) prove that the performance of the gyroscope under a mode-matching condition is improved. When the method of open-loop detection with frequency tuning, compared with the method of open-loop detection without frequency tuning, was employed, the scale factors were increased by 19.8 times, 10.7 times, and 9.4 times, the ARW was improved by 157%, 147% and 156%, and the zero bias stability was promoted by 3.26%, 4.09% and 17.08% at −20 ${}^{\circ }$C, room temperature, and 60 ${}^{\circ }$C, respectively. In addition, the method of closed-loop detection by frequency tuning was adopted, and, compared with the method of open-loop detection with frequency tuning, the scale factor nonlinearity and bandwidth under the premise of maintaining the same static performance improved. However, the large damping coupling and the small quality factor resulted in a large drift of the gyroscope static output, which made the improvement of the zero bias stability in the mode-matching condition not obvious. Moreover, the structure of quadrature correction combs cannot respond to higher frequency sinusoidal signals, thereby limiting the working bandwidth of the gyroscope. Therefore, it is necessary to design a quadrature stiffness correction comb that can respond to a higher frequency sinusoidal input signal that improves the operating bandwidth limitations of the gyroscope. The quality factor of the gyroscope needs to be improved, and an effective compensation method can be adopted to suppress the zero bias drift caused by the small quality factor and damping coupling, thereby improving the static performances of the gyroscope. 

## Figures and Tables

**Table 1:** Simulation parameters of the dual-mass MEMS gyroscope.

Parameter	Values	Units
Drive mode resonant frequency ($\omega_{x}$)	3925.29 × 2π	rad/s
Drive mode quality factor ($Q_{x}$)	4673	
Sense mode resonant frequency ($\omega_{y}$)	3978.45×2 π	rad/s
Sense mode quality factor ($Q_{y}$)	449	
Drive effective mass ($m_{x}$)	$1.42\times 10^{-6}$	kg
Sense effective mass ($m_{y}$)	$1.58\times 10^{-6}$	kg
Drive mode capacitance ($C_{d}$)	2.88	pF
Sense mode capacitance ($C_{d}$)	4.68	pF
Quadrature correction comb number ($n_{q}$)	30	
Quadrature correction comb thickness ($h_{q}$)	60	um
Quadrature correction comb gap ($d_{q}$)	5	um
Comb overlap length ($l_{q}$)	10	um
Unequal spacing ratio (λ)	2.5	
Vacuum permittivity ($\varepsilon_{0}$)	$8.85\times 10^{-12}$	F/m
Tuning comb number ($n_{t}$)	300	
Tuning comb number thickness ($h_{t}$)	60	um
Correction comb gap ($d_{t}$)	4	um
Comb overlap length ($l_{t}$)	200	um
Low-frequency signal amplitude ($A_{t}$)	1	
Input signal frequency ($\omega_{t}$)	2π×80	rad/s
DC benchmark voltage ($V_{d}$)	2.048	V
Interface circuit amplification factor ($K_{pre}$)	$7.6159\times 10^{7}$	
Controller parameters ($K_{p}$)	30	
Controller parameters ($K_{i}$)	0.0075	
Reference voltage ($V_{ref}$)	0.2587	mV

**Table 2:** Scale factor performance of the tested gyroscope.

Temperature	Test Type	Scale Factor	Scale Factor Nonlinearity	Scale Factor Asymmetry
		(mV/${}^{\circ }$/s)	(ppm)	(ppm)
−20 ${}^{\circ }$C	Test1	−2.039	142	546
	Test2	40.453	107,155	46,978
	Test3	3.857	53	212
60 ${}^{\circ }$C	Test1	−2.048	144	596
	Test2	19.324	2526	10,074
	Test3	3.780	96	568
Room temperature	Test1	−2.051	322	1009
	Test2	22.024	3398	14,792
	Test3	3.786	28	128

**Table 3:** Static performance of the three test methods at different temperatures.

Temperature	Test Type	Zero Bias (${}^{\circ }$/s)	Zero Bias Stability (${}^{\circ }$/h)	ARW (${}^{\circ }/\sqrt{h}$)
−20 ${}^{\circ }$C	Test1	1.138	24.746	12.780
	Test2	5.554	23.964	4.966
	Test3	5.381	24.580	4.862
60 ${}^{\circ }$C	Test1	5.991	70.187	12.536
	Test2	13.127	67.429	5.085
	Test3	13.526	64.926	5.058
Room temperature	Test1	1.458	43.439	12.229
	Test2	2.602	37.648	4.784
	Test3	2.601	39.545	4.956
